# The size, number and bilaterality of endometriomas do not affect spontaneous conception chance following surgical removal

**DOI:** 10.5935/1518-0557.20220067

**Published:** 2023

**Authors:** Şeyma Osmanlıoğlu, Yavuz Emre Şükür, Koray Görkem Saçıntı, Batuhan Özmen, Murat Sönmezer, Bülent Berker, Ruşen Aytaç, Cem Somer Atabekoğlu

**Affiliations:** 1 Ankara Medipol University Faculty of Medicine, Department of Gynaecology and Obstetrics, Ankara, Turkey; 2 Ankara University Faculty of Medicine, Department of Gynaecology and Obstetrics, Ankara, Turkey

**Keywords:** endometrioma, endometriosis, fertility, spontaneous conception, surgery

## Abstract

**Objective:**

Endometrioma surgery is associated with a reduction in ovarian reserve. However, removal of an endometrioma may increase the likelihood of a spontaneous conception. The objective of this study was to assess the pre-operative and operative variables affecting spontaneous conception following endometrioma surgery.

**Methods:**

Data from 211 women ≤40 years of age who underwent an endometrioma surgery at a university-based infertility clinic between January 2005 and June 2020 were reviewed retrospectively. The main outcome measure was spontaneous clinical pregnancy. We had 84 women with and 127 women without a successful spontaneous conception making up the case and control groups.

**Results:**

The median ages of the cases and controls were 27 and 32 years, respectively (*p*<0.001). The rate of recurrence was significantly lower in the spontaneous conception group when compared to controls (29.8% vs. 52.8%, respectively; *p*=0.001). Our results showed no differences in the number, size, or side of the endometriomas in both groups. Multivariate logistic regression analysis showed significant independent effects of age (B: -.166, OR {odds ratio}: 0.847, 95% CI {confidence interval}: 0.791-0.907, *p*<0.001), recurrence (B: -1.030, OR: 0.357, 95% CI: 0.188-0.678, *p*=0.002), and laparoscopic surgery rather than laparotomy (B: 1.585, OR: 4.879, 95% CI: 1.029-23.133, *p*=0.046) for spontaneous conception.

**Conclusions:**

The size, number and bilaterality of the endometrioma did not affect the spontaneous conception likelihood following surgical removal. However, increasing age and recurrence are negatively associated with the likelihood of spontaneous conception. Laparoscopic surgery may increase the chance of spontaneous conception when compared to laparotomy.

## INTRODUCTION

Endometriosis is an estrogen-dependent, benign, chronic inflammatory disease with ectopic endometrial implants (Macer & Taylor, 2012), that affects at least 4% of women in reproductive age (Ferrero *et al*., 2010). Endometrioma is a typical manifestation of the ovarian disease, and its prevalence ranges from 17 to 44% of patients with endometriosis (Busacca & Vignali, 2003).

To what extent the endometrioma and endometrioma surgery influence the ovarian reserve and spontaneous ovulation is controversial. It has been claimed that women with endometriomas have lower levels of anti-Müllerian hormone (AMH) and antral follicle counts (AFC) compared to women without ovarian cysts, suggesting that the presence of endometrioma is associated with a reduction in ovarian reserve (Uncu *et al*., 2013). On the other hand, a prospective observational study showed that endometriomas, irrespective of their volume, do not influence the rate of spontaneous ovulation in the affected ovary; furthermore, a good spontaneous pregnancy rate was demonstrated if the couple had no other risk factor for infertility (Leone Roberti Maggiore *et al*., 2015).

The majority of publications show that surgery does not increase the success rate of in vitro fertilization (IVF) and that it may harm the assisted reproductive technology (ART) resulted by reducing the ovarian response to controlled ovarian stimulation (COH) (Gupta *et al*., 2006; Hamdan *et al*., 2015; Kuroda *et al*., 2012). Besides, several studies have reported a reduced AMH after endometrioma surgery (Cranney *et al*., 2017; Somigliana *et al*., 2012; Streuli *et al*., 2012), while antral follicle count seems to be comparatively less affected (Muzii *et al*., 2014). On the other hand, some studies showed recovery of the ovarian reserve after endometrioma surgery for up to one year (Chang *et al*., 2010; Iwase *et al*., 2016), so that spontaneous conception can be expected one year after surgery.

Since the first description of the Endometriosis fertility index (EFI), predictability of the best fertility treatment for women with endometriomas received more attention in recent decades (Adamson & Pasta, 2010). To further investigate this topic, we conducted a retrospective analysis examining the spontaneous conception rate after endometrioma surgery. The aim of this study was to assess the impact of pre-operative and operative characteristics on future fertility likelihood in women who underwent endometrioma surgery.

## MATERIALS AND METHODS

In the present retrospective cohort study, we reviewed data from women with the histological diagnosis of endometriosis who underwent surgery at a university-based infertility clinic between January 2005 and June 2020. The study was approved by the Clinical Research Ethical Committee of Ankara University School of Medicine (Approval no: 15-775-16). The patient data and the follow-up information (up to 12 months) were extracted from the medical records. Inclusion criteria consisted of (1) histologically diagnosed uni/bilateral endometrioma(s), (2) women ≤40 years, (3) women with regular unprotected intercourse. Exclusion criteria were as follows: (1) histologically diagnosed endometriosis without uni/bilateral endometrioma, (2) postmenopausal status at the time/after the operation, (3) women >40 years, (4) women without follow-up information, (5) women with contraception, (6) women with other uncorrected gynecological problems such as leiomyomas or uterine abnormalities. All infertile patients were assessed for tubal patency using hysterosalpingogram (HSG) before surgery and/or chromopertubation at surgery. In addition, patients who underwent surgery because of pain and had future fertility plans were also assessed for tubal patency using chromopertubation at the surgery. Hence, the patients with documented bilateral tubal obstruction were excluded from the study. Besides, semen analysis was also performed for all infertility patients and the patients with male factor infertility were excluded from the study.

All patients included in the study underwent either a laparotomy or a laparoscopic surgery by the same experienced team under general anesthesia with a standard technique. Laparoscopy was performed in almost all patients, and a laparotomy only in clinically indicated cases. During laparoscopy, the cyst wall was detached from the healthy surrounding ovarian tissue with two atraumatic grasping forceps by traction and countertraction after identifying the cleavage plane. If necessary, hemostasis was achieved with bipolar forceps, which were used as little as possible to avoid damaging healthy tissue. In the laparotomy, the cyst wall was removed by hand using an atraumatic forceps, and hemostasis was performed with bipolar forceps. The operation was indicated for uni/bilateral endometrioma(s) detected by ultrasound with accompanying symptoms (either pain symptom or infertility). For the multiple endometriomas, the size of all endometriomas calculated together was determined as the endometrioma size. The main outcome measure was spontaneous clinical pregnancy within twelve months following surgery, that was defined as the presence of a fetus with a heartbeat at 6 weeks of gestation. In addition, demographics were compared between the women who got pregnant spontaneously and those who could not.

### Statistical Analyses

Data analyses were performed by using the SPSS Version 21.0 (IBM Corporation, Armonk, NYC, USA). The samples were tested using the Kolmogorov-Smirnov test to determine normality of distribution. According to the results, non-parametric tests were preferred. Continuous variables were compared using the Mann-Whitney U test and the categorical variables were compared using the Chi-square test or the Fisher’s exact test, where appropriate. Multivariate logistic regression analyses with a model building strategy were used to determine independent predictors of spontaneous conception following endometrioma surgery. Variables included in the model were age, unilateral salpingectomy, unilateral oophorectomy, type of operation, and endometrioma recurrence. A *p* value of <0.05 was considered statistically significant.

## RESULTS

**Figure 1:**
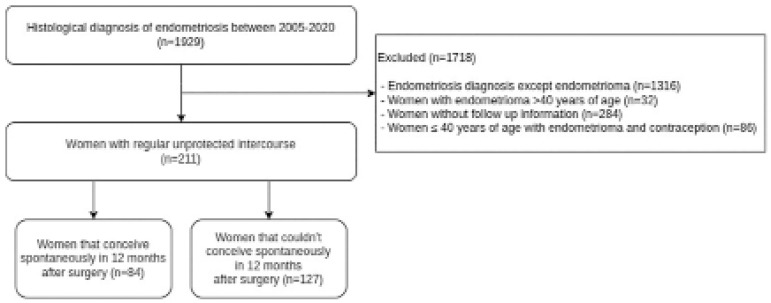
Flow diagram of the study population.

Of a total of 1929 histologically diagnosed endometriosis cases, 1718 patients were excluded since they didn’t meet the inclusion criteria of the study. A total of 211 women with uni- or bilateral endometriomas were included in the final analyses. Figure 1 summarizes the flow diagram of the study population. Eighty-four women with spontaneous conception formed the case group and 127 women without successful spontaneous conception the control group. The median age of the case group was 27 years and the control group 32 years, respectively (*p*<0.001). Tables 1 and 2 show the demographic data of the study population as well as the comparison of various parameters between women that could and couldn’t conceive spontaneously.

**Table 1:** Demographics of the study population.

	Cases N=84	Controls N=127	*p*
Age, years	27 (24-30)	32 (27-36)	<0.001
BMI, kg/m^2^	22.6 (20.9-24.8)	22.6 (20.3-25.9)	0.68
Endometrioma size, cm	5 (4-6.75)	5 (4-7)	0.697
Duration of infertility before surgery (y)	0 (0-2)	0.5 (0-3)	0.137
No. of pregnancies before surgery	0 (0-1)	0 (0-1)	0.077

BMI: body mass index. Values are presented as median (25p-75p).

**Table 2:** Comparison of the endometrioma related features in women with and without a successful spontaneous conception.

	Cases N=84	Controls N=127	*p*	OR	95% CI
Endometrioma side, n (%) Unilateral Bilateral	50 (59.5) 34 (41.5)	73 (57.5) 54 (42.5)	0.768		
Endometrioma multilocular, n (%)	6 (7.1)	6 (4.7)	0.458		
Additional operation, n (%) None Myomectomy Other uterine or tubal operations	65 (77.4) 8 (9.5) 11 (13.1)	100 (78.7) 16 (12.7) 10 (7.9)	0.404		
Unilateral oophorectomy, n (%)	0	12 (9.4)	0.002		
Unilateral salpingectomy, n (%)	1 (1.2)	15 (11.8)	0.003	0.613	0.514-0.730
Adhesions, n (%)	30 (35.7)	56 (44.1)	0.225		
Douglas obliteration, n (%)	13 (15.5)	27 (21.3)	0.294		
Type of the operation, n (%) Laparoscopy Laparotomy	77 (97.5) 2 (2.5)	97 (84.3) 18 (15.7)	0.002	4.293	1.141-16.150
Smoking, n (%)	23 (27.4)	45 (35.4)	0.221		
Preoperative pain symptom, n (%)	64 (76.2)	100 (78.7)	0.663		
Preoperative infertility, n (%)	35 (42.2)	66 (52)	0.165		
Recurrence, n (%)	25 (29.8)	67 (52.8)	0.001	0.692	0.557-0.861
Second operation after recurrence, n (%)	4 (16)	23 (34.4)	0.122		
Postoperative add-back therapy, n (%) None COCs GnRH-analogues LNG-IUS	53 (63.1) 16 (19) 15 (17.9) 0 (0)	74 (58.3) 37 (29.1) 15 (11.8) 1 (0.8)	0.240		

OR: odds ratio; 95% CI: 95% confidence interval; COC: combined oral contraceptive; GnRH: gonadotrophin releasing hormone; LNG-IUS: levonorgestrel releasing intrauterine system.

Fifty patients (59.5%) in the case group and seventy-three patients (57.5%) in the control group had a unilateral endometrioma. On the other hand, thirty-four (41.5%) in the case group and fifty-four patients (42.5%) in the control group had bilateral endometriomas (*p*=0.768). Six patients in the case group (7.1%) and six patients (4.7%) in the control group had multilocular endometriomas (*p*=0.458). Twenty-five (29.8%) patients in the case group and sixty-seven (52.8%) in the control group had recurrences (>1cm in diameter) detected by ultrasound (*p*=0.001). Due to endometrioma recurrence, four patients (16%) in the case group and twenty-three patients (34.4%) in the control group were operated for a second time (*p*=0.122). One patient (1.2%) in the case and fifteen patients (11.8%) in the control group had a history of unilateral salpingectomy or had a salpingectomy during the operation (*p*=0.003). Both ovaries were present in all patients in the case group and twelve patients (9.4%) in the control group had a history of unilateral oophorectomy or had an oophorectomy during the operation (*p*=0.002). The median (range) of previous pregnancies was 0 (0-1) in both groups (*p*=0.077). Thirteen patients (15.5%) in the case group and twenty-seven patients (21.3%) in the control group had evidence of deep endometrioses (*p*=0.29), although a detailed classification was missing in many surgical reports. Sixty-four (76.2%) of the patients in the case group and one hundred (78.7%) of the patients in the control group had chronic cyclic pain before surgery (*p*=0.663). Most women in both groups underwent laparoscopy, with the laparoscopy rate being significantly higher in the case group (97.5% *vs*. 84.3%; *p*=0.002). Additional operations, such as myomectomy and other uterine or tubal operations have been also considered but didn’t show any difference in both groups (Table 2).

Most of the patients in both groups did not receive any hormonal add-back therapy after the operation [case group n=53 (63.1%); control group n=74 (58.3%); *p*=0.24]. Postoperative use of combined oral contraceptives (COCs) for three months was ordered for 16 patients (19%) in the case group and for 37 patients (29.1%) in the control group. A subcutaneous injection of GnRH-analogues was given to 15 patients (17.9%) in the case group and to 15 patients (11.8%) in the control group. Only in one patient in the control group, a levonorgestrel-releasing intrauterine system (LNG-IUS), Mirena^®^ was inserted for three months. A multivariate regression analysis showed a significant independent effect of age, recurrence of endometrioma, and type of operation and did not suggest a significant effect of unilateral salpingectomy (Table 3).

**Table 3:** Logistic regression for the chance of obtaining spontaneous conception among women, that tried to conceive.

	B	OR	95% CI	*p*
Age	-.166	0.847	0.795-0.913	<0.001
Recurrence	-1.030	0.357	0.188-0.678	0.002
Operation type (Laparoscopy)	1.585	4.879	1.029-23.133	0.046
Unilateral salpingectomy	-1.184	0.306	0.630-1.491	0.072

OR: odds ratio; 95% CI: 95% confidence interval. Backward stepwise logistic regression analysis was performed (Hosmer & Lemeshow goodness of fit test: Chi-square: 2.602, df: 8, *p*=0.957).

## DISCUSSION

Our study showed a significant independent effect of age and endometrioma recurrence on spontaneous conception after endometrioma surgery. The patients in our case group were younger and the recurrence rate was less than that in the control group. There was no significant difference between the two groups regarding the side, number and size of the endometrioma cyst, smoking, BMI, preoperative pain symptom and preoperative pregnancy rate.

Since the incidence of endometrioma increases with age, and family planning being postponed to older ages, the issue of endometrioma related to fertility has received more attention in recent decades. Oocyte quality is known to have direct effects on ART success (de Ziegler *et al*., 2019). The effect of endometriosis on oocyte quality, on the other hand, is controversial. Several studies claim that especially advanced stage endometriosis negatively affects oocyte quality (Brosens, 2004; Hauzman *et al*., 2013), while several others show the opposite (González-Comadran *et al*., 2017; Juneau *et al*., 2017). A diminished ovarian reserve (DOR) reflects a decrease in the number and quality of oocytes, which currently is the second leading cause of infertility (Buyuk *et al*., 2011). Although it can arise from a variety of factors, DOR is mainly caused by advanced maternal age. In the present study the maternal age was one of the main factors of successful conception. The women with endometriomas who could conceive spontaneously were significantly younger than those who could not conceive spontaneously (median 27 *vs*. 32 years; *p*<0.001). The median time between endometrioma surgery and spontaneous conception was less than one-year (median (Interquartile Range (IQR)): 6 (2-10) months). In this study, the women without success in spontaneous conception were not examined for the other causes of infertility. If the other causes of infertility could be ruled out, the ratio of successful spontaneous conception could increase. On the other hand, the higher rate of successful conception can be explained by the fact that some of the patients did not have a history of infertility and our cohort consisted of relatively young patients.

In a prospective analysis of AMH levels in women undergoing surgery, there was no difference between endometrioses patients at all stages and controls, but levels were lower in women who had previously undergone endometrioma surgery (Streuli *et al*., 2012). The risk of reduced ovarian reserve after endometriosis surgery occurs especially in the presence of large (> 7 cm), bilateral endometriomas, as well as the surgical removal of multiple endometrioma cysts (Chen *et al*., 2014; Cranney *et al*., 2017; Hamdan *et al*., 2015; Streuli *et al*., 2012). Causes of ovarian damage during endometrioma surgery include mechanical damage associated with removal of healthy ovarian tissue along with the cyst wall, and heat damage produced by the energy modalities used during hemostasis after cyst removal, especially if the operation is performed by a surgeon with limited experience (Muzii *et al*., 2011). In addition, it has been shown that the age of menopause is significantly lower in women who have undergone previous endometrioma surgery compared to the normal population (Coccia *et al*., 2011). On the other hand, some studies showed recovery of the ovarian reserve after endometrioma surgery up to one year in reproductive women (Chang *et al*., 2010; Iwase *et al*., 2016). Our results showed no differences in the number, size, or side of the endometriomas in both groups. Contradictory findings in the literature and the high pregnancy rate found in the current study after an endometrioma surgery suggests that, despite the decrease in ovarian reserve after endometrioma surgery, it could be restored thereafter up to one year postoperative. Therefore, favorable preoperative ovarian reserve and an operation performed by a surgeon with high experience may implicate a postsurgical pregnancy after endometrioma surgery.

Our results indicate that, women without endometrioma recurrence are significantly more likely to get spontaneously pregnant. Furthermore, a second surgery after recurrence of endometrioma seemed to decrease the likelihood of spontaneous conception. European Society of Human Reproduction and Embryology (ESHRE) guidelines recommend a cystectomy rather than CO2 laser vaporization in women with ovarian endometrioma, because of a lower recurrence rate of the endometrioma (Dunselman *et al*., 2014). The reduced conception that we found in patients with recurrent endometriomas and the recommendation of ESHRE indicating that, a cystectomy should be performed by an experienced surgeon during the first operation.

Laparoscopy is the gold standard for diagnosing endometriosis and also provides for an opportunity for treatment (Nezhat *et al*., 2022). The majority of women in both groups underwent laparoscopy, with the laparoscopic rate being significantly higher in women with successful spontaneous conception. In addition, laparoscopic surgery rather than laparotomy independently increased the likelihood of spontaneous conception after surgery. This could be related to the higher stage of endometriosis and the associated laparotomy indication and fertility risk. Cyst stripping and electrocoagulation of the cyst wall was the only method used in all operations. A unilateral salpingectomy reduced the success of spontaneous conception (11.8% *vs*. 1.2%; *p*=0.003), as well as a unilateral oophorectomy (0 *vs*. 9.4%; *p*=0.003), whereby no significant influence of adhesions and Douglas obliteration could be demonstrated. A limiting factor for this evaluation was the inaccurate classification of the endometriosis in the operation reports, so that it is difficult to conclude regarding the endometriosis stage and adhesions.

Although postoperative hormone therapy could have some effects on the success rate of spontaneous conception, we could not evaluate it in this study, since both groups were relatively similar vis-a-vis the hormonal treatment.

Our study may have some limitations. The retrospective design implies a lower level of evidence for the conclusions. The lack of a determination of ovarian reserve was also a limitation of the present study. Since our patients were initially diagnosed with endometrioma independent of their fertility status, there was not enough data to evaluate the ovarian reserve whether with AMH or with AFC. In ART, AMH can be used to predict the ovarian response to gonadotrophin stimulation. However, it has only a minor influence on the likelihood of achieving natural conception (Hamdine *et al*., 2015; La Marca *et al*., 2010). In addition, pre-operative AMH concentration is increased in women with endometriomas, especially with a cyst size of over 6 cm (Marcellin *et al*., 2019; Roman *et al*., 2021), so that AFC was recommended as a marker for the ovarian reserve in contrast to AMH in women with endometriomas (González-Foruria *et al*., 2020). One of the major limitations of this study was lack of an accurate classification of endometriosis in the operation reports, since information on endometriosis stage and adhesion is very important to relation to the subject of this study. Another limitation of the study was that the male factor was only examined in the infertility patients and not in the patients who underwent surgery because of pain. The lack of a control group of women without endometrioses was another limitation. Nonetheless, the lack of a control group did not affect the design, as the comparisons between women with and without success of spontaneous conception were evaluated.

All in all, despite its its limitations, the current study suggests that the maternal age and endometrioma recurrence may have major influence on the success rate of spontaneous conception for endometrioma patients after endometrioma surgery. A favorable preoperative ovarian reserve, better determined with AFC, and a cystectomy performed by an experienced surgeon may lead to postoperative pregnancy after endometrioma surgery. Future prospective studies are required to assess the spontaneous pregnancy rate after endometrioma surgery.
